# Trends in cause-specific mortality among children aged 5–14 years from 2005 to 2016 in India, China, Brazil, and Mexico: an analysis of nationally representative mortality studies

**DOI:** 10.1016/S0140-6736(19)30220-X

**Published:** 2019-03-16

**Authors:** Shaza A Fadel, Cynthia Boschi-Pinto, Shicheng Yu, Luz Myriam Reynales-Shigematsu, Geetha R Menon, Leslie Newcombe, Kathleen L Strong, Qiqi Wang, Prabhat Jha

**Affiliations:** a Centre for Global Health Research, St Michael's Hospital, Dalla Lana School of Public Health, University of Toronto, Ontario, Canada; b Instituto de Saude Coletiva, Universidade Federal Fluminense, Niteroi, Brazil; c Chinese Center for Disease Control and Prevention, Beijing, China; d Instituto Nacional de Salud Publica, Cuernavaca, Mexico; e Indian Council of Medical Research, Ansari Nagar, New Delhi, India; f Maternal, Newborn, Child and Adolescent Health Department, WHO, Geneva, Switzerland

## Abstract

**Background:**

With global survival increasing for children younger than 5 years of age, attention is required to reduce the approximately 1 million deaths of children aged 5–14 years occurring every year. Causes of death at these ages remain poorly documented. We aimed to explore trends in mortality by causes of death in India, China, Brazil, and Mexico, which are home to about 40% of the world's children aged 5–14 years and experience more than 200 000 deaths annually at these ages.

**Methods:**

We examined data on 244 401 deaths in children aged 5–14 years from four nationally representative data sources that obtained direct distributions of causes of death: the Indian Million Death Study, the Chinese Disease Surveillance Points, mortality data from the Mexican Instituto Nacional de Estadística y Geografía, and mortality data from the Brazilian Institute of Geography and Statistics. We present data on 12 main disease groups in all countries, with breakdown by communicable and nutritional diseases, non-communicable diseases, injuries, and ill-defined causes. To calculate age-specific and sex-specific death rates for each cause, we applied the national cause of death distribution to the UN mortality envelopes for 2005–16 for each country.

**Findings:**

Unlike Brazil, China, and Mexico, communicable diseases still account for nearly half of deaths in India in children aged 5–14 years (73 920 [46·1%] of 160 330 estimated deaths in 2016). In 2016, India had the highest death rates in nearly every category, including from communicable diseases. Fast declines among girls in communicable disease mortality narrowed the gap by 2016 with boys in India (32·6 deaths per 100 000 girls *vs* 26·2 per 100 000 boys) and China (1·7 *vs* 1·5). In China, injuries accounted for the greatest proportions of deaths (20 970 [53·2%] of 39 430 estimated deaths, in which drowning was a leading cause). The homicide death rate at ages 10–14 years was higher for boys than for girls in Brazil, increasing annually by an average of 0·7% (0·3–1·1). In India and China, the suicide death rates were higher for girls than for boys at ages 10–14 years. By contrast, in Mexico it was higher for boys than for girls, increasing annually by an average of 2·8% (2·0–3·6). Deaths from transport injuries, drowning, and cancer are common in all four countries, with transport accidents among the top three causes of death for both sexes in all countries, except for Indian girls, and cancer in the top three causes for both sexes in Mexico, Brazil, and China.

**Interpretation:**

Most of the deaths that occurred between 2005 and 2016 in children aged 5–14 years in India, China, Brazil, and Mexico arose from preventable or treatable conditions. This age group is important for extending some of the global disease-specific targets developed for children younger than 5 years of age. Interventions to control non-communicable diseases and injuries and to strengthen cause of death reporting systems are also required.

**Funding:**

WHO and the University of Toronto Connaught Global Challenge.

## Introduction

In 2016, there were approximately 0·7 billion children younger than 5 years of age and 1·2 billion children aged 5–14 years globally.^1^ Global death totals for children aged 5–14 years are about one sixth of those of younger children and death rates among children at these ages are among the lowest during the human lifespan, but they are not insubstantial.^2^ Recently, attention has focused on better quantification of the death rates and causes for children aged 5–14 years. Hill and colleagues, using country-level survey data, estimated 1·5 million deaths among children aged 5–14 years in 2010.^3^ The UN Inter-agency Group for Child Mortality Estimation (IGME) estimated that about 1 million children aged 5–14 years died in 2017, whereas the UN World Population Prospects (WPP) estimated this number as 1·2 million.^1^, ^2^ By contrast, the model-based Global Burden of Diseases, Injuries, and Risk Factors Study (GBD) estimated 0·7 million deaths in children aged 5–14 years in 2017.^4^

**Research in context**

**Evidence before this study**

We searched MEDLINE, Embase, and CABI Global Health for studies on causes of death for children aged 5–14 years in India, China, Brazil, and Mexico published from Jan 1, 2005, to Oct 15, 2018, with no language restrictions. From 1252 articles screened, 15 articles reported a distribution of at least three causes of death and used nationally representative data. Most studies examined only one or a few selected causes. This includes pneumonia and diarrhoea in the Million Death Study using data from 2001 to 2003; injuries in China; five causes aggregated to ages 5–19 years in Mexico; and changing causes of death in Brazil. The Global Burden of Disease project and WHO produce modelled estimates for the four countries.

**Added value of this study**

We provide direct evidence on cause-specific time trends for deaths of children aged 5–14 years for India, China, Brazil, and Mexico using nationally representative cause of death data, accounting for about 40% of the population in this age group. Marked declines in communicable disease mortality among girls have narrowed the gap with boys in India and China. Mortality rates for non-communicable disease and injury are still higher in India than in China, Mexico, and Brazil. By contrast with Mexico and Brazil, a majority of road traffic deaths in both India and China were among pedestrians. Most child homicides in Brazil and Mexico were firearm related and suicide mortality is increasing for boys in Mexico. Despite regional differences, the common leading causes of death in all four countries include transport accidents, drowning, cancers, and neurological diseases.

**Implications of all the available evidence**

Analysis of direct cause-specific mortality among children aged 5–14 years provides clear targets for prevention and treatment of the conditions causing more than 200 000 deaths annually at these ages in India, China, Brazil, and Mexico. Effective interventions to reduce child mortality exist and would need to go beyond the maternal and child health interventions for younger children. Improved coverage and quality of direct mortality data are needed to measure progress in reducing these deaths, and to decrease reliance on the more uncertain modelled estimates.

GBD and WHO cause of death reports before 2005 substantially underestimated deaths from infections and their sequelae—notably pneumonia, malaria, and diarrhoea—among children aged 5–14 years.^5^, ^6^ More recent reports have benefited from direct mortality data from earlier rounds of the Million Death Study (MDS) in India and from improved availability of vital registration data from China and verbal autopsy studies in Africa.^7^ Moreover, the cause of death distribution suggests that some relevant global health targets, including the 2030 UN Sustainable Development Goals (SDGs), might consider children up to age 15 years and not only younger than 5 years. This consideration would require a greater understanding of the causes of death among children aged 5–14 years, ideally from direct evidence versus more uncertain modelling. India, China, Brazil, and Mexico are home to about 40% of the world's children aged 5–14 years and experience about a quarter of global mortality at these ages.^2^ Here, we provide direct estimates of the trends and levels of cause-specific mortality among children aged 5–14 years in these four countries from 2005 to 2016, using nationally representative data. We discuss these results in the context of global efforts to reduce under-5 and under-15 mortality.

## Methods

### Data sources

The choice of countries was based on a WHO consultation and review of available data sources that provided cause of death for children aged 5–14 years worldwide. After excluding high-income countries with low death rates, we examined 20 low-income countries and 19 middle-income countries with the highest number of deaths among children aged 5–14 years. Collectively, these countries comprised about 88% of all deaths for children aged 5–14 years in 2017 (appendix). For inclusion, we required availability of at least 10 years of nationally representative data with sufficient quality. South Africa, the Philippines, and Egypt did not meet our inclusion criteria and the remaining countries did not have nationally representative mortality data. The four remaining countries represented 203 272 (87%) of the 233 651 deaths in 2017 in the seven middle-income countries originally considered. We used four nationally representative data sources that obtained direct (*vs* modelled) distributions of causes of death: the Indian MDS, the Chinese Disease Surveillance Points (DSP), mortality data from the Mexican Instituto Nacional de Estadística y Geografía, and mortality data from the Brazilian Institute of Geography and Statistics. The details of each have already been published.^8^, ^9^, ^10^

Briefly, in collaboration with the Registrar General of India, the MDS monitored approximately 14 million people in 2·4 million nationally-representative households in India from 1998 to 2013. One of 900 trained non-medical surveyors implemented a well validated verbal autopsy (based on the 2012 WHO instrument) for each death that occurred in these households during the preceding 6 months. Two of 400 trained physicians assigned a cause of death.^8^ The Chinese Center for Disease Control and Prevention coordinates the China DSP, a sample-based mortality surveillance system that uses a narrative-based verbal autopsy and has been nationally representative since 2004. The DSP system was integrated with the Chinese Vital Registration System in 2013.^9^ The DSP expanded from 161 sites in 2004 to 605 sites covering 324 million people in urban and rural areas in 2013.^9^ Local physicians or central doctors assign the cause of death. From 2004 to 2012, the DSP redistributed ill-defined codes (accounting for <2% of cause-specific mortality [range 1·3–2·0]) to other specific International Classification of Diseases 10th revision (ICD-10) codes.^11^ More than 95% of deaths in Mexico are registered and less than 2% of the causes are ill defined.^10^ More than 90% of deaths are registered in Brazil, albeit with regional variation in quality.^10^ We excluded 19 deaths (0·02%) where sex was not reported from Brazil. Data from 2014–16 are not yet available from the Indian MDS and deaths for 2011 were not available for China.

All mortality data were available at the individual level except for deaths from China, which were aggregated by 5-year age groups and sex and grouped into ICD-10 codes. We classified deaths using a modified ICD-10 classification list (appendix). The cause of death classification list captured 27 distinct causes across the four countries. To improve the stability of the trends, we present data in 12 main disease groups relevant to the SDG goals and broken down into communicable and nutritional, non-communicable diseases (NCDs), injuries, ill defined, and overall mortality. We retained ill-defined codes as a measure of quality in all countries (China from 2013).^12^

### Death rate calculations

Cause of death data from India and China are based on random samples. For India, we calculated the cause distribution annually, weighted by the sampling probability for rural and urban areas of 35 states and union territories, as described previously.^13^ We extrapolated forward to 2016 using moving averages of deaths and the trends from 2001–13. For China, we calculated death rates using the number of sample deaths divided by their sample population for each rural and urban area separately. We interpolated for 2011 on the basis of prior year trends for China. We calculated age-specific and sex-specific death rates nationally using deaths from Mexico and Brazil's vital registration systems and mid-year age-specific populations from the UN for each year. For each sex and 5-year age group, we applied the national cause of death distribution smoothed by 3-year forward-moving averages from the sample (China and India) or vital registration (Mexico and Brazil) systems to the IGME total mortality envelopes for 2005–16 for each country.^2^ The IGME has not yet published the sex-specific mortality for children older than 5 years for 2017. Thus, we applied the sex ratios from interpolated annual death rates from the WPP to the IGME's death rates to get age-specific and sex-specific death rates (appendix).^1^ The use of the UN totals has the additional benefit of correcting for the undercounts of total deaths in the country estimates because the UN mortality envelopes add a correction for possible undercounts. The Indian SRS undercounts deaths in people older than 5 years for about 1–4% in males and 8–11% in females.^1^ For China, the DSP undercounts up to 16% of deaths for children aged 6–14 years.^14^

We standardised age-specific death rates using the WHO standard population (with weights of 0·5026 for age 5–9 years and 0·4974 for age 10–14 years). To calculate peak firearm-related mortality for homicide and suicide deaths in Brazil and Mexico, we used crude death rates without any smoothing by moving averages. We calculated the average annual rate of reduction in the death rates as described elsewhere^15^ and present them with 95% CIs. We used Stata 15.1 for statistical analyses.

### Role of the funding source

The sponsors of the study had no role in the study design, data collection, data analysis, or data interpretation. The corresponding author had full access to all the data in the study and had final responsibility for the decision to submit for publication.

## Results

We analysed 244 401 deaths in children aged 5–14 years occurring between 2005 and 2016 in India, China, Brazil, and Mexico (table 1). Death rates from most of the wider cause-of-death groupings declined from 2005 to 2016 in both sexes in all countries. India had the fastest decline of any country for both sexes and with faster annual declines in communicable causes of death for girls than for boys (table 1; appendix). Despite the rapid declines, age-standardised death rates from communicable diseases in 2016 were about 17–19 times greater in India than in China and about 7–12 times greater than in Brazil or Mexico, depending on sex. Thus India was the only country where communicable diseases still account for nearly half of deaths. Starting from a much lower absolute level of mortality, communicable diseases in China also fell more rapidly in girls than in boys (table 1). The rates of decline in NCD mortality were broadly similar in India and in China but NCDs rose modestly in girls in Brazil and rates of decline were also modest in Brazilian boys and in Mexican girls and boys. The proportion of ill-defined deaths was generally low: less than 0·8% in China and in Mexico, 2·4% in India, and 5·6% in Brazil (with these proportions decreasing over time).

**Table 1:** Study size and age-adjusted death rate for children aged 5–14 years in India, China, Brazil, and Mexico

	**Study deaths (n=244 401)**	Age-standardised death rate per 100 000 population*				**Annual reduction (95% CI), 2005–16**†	
		Girls‡		Boys‡		Girls‡	Boys‡
		2005	2016	2005	2016		
**India**							
Communicable or nutritional§	5239	82·6	32·6	57·5	26·2	8·7% (8·2 to 9·1)	7·4% (6·9 to 7·8)
Non-communicable	2050	20·9	13·4	19·7	13·8	4·4% (2·9 to 5·9)	3·3% (2·4 to 4·2)
Injuries	2580	22·7	15·8	30·2	20·8	2·3% (1·6 to 3·1)	2·4% (1·6 to 3·3)
Ill defined	240	2·8	2·3	2·3	1·9	0·0% (−2·6 to 2·6)	3·4% (1·2 to 5·4)
All causes¶	10 109	129·0	64·1	109·7	62·6	6·3% (6·1 to 6·5)	5·0% (4·9 to 5·0)
**China**							
Communicable or nutritional§	3160	4·4	1·7	3·0	1·5	8·9% (7·3 to 10·6)	5·2% (3·7 to 6·7)
Non-communicable	14 887	11·8	10·2	10·4	8·9	2·3% (1·1 to 3·4)	1·6% (0·4 to 2·8)
Injuries	24 191	18·0	10·4	24·8	15·1	4·2% (3·6 to 4·8)	4·4% (4·0 to 4·8)
Ill defined (2013–16)||	331	··	0·3	··	0·3	21·8% (8·6 to 33·1)	24·8% (11·7 to 36·0)
All causes	42 569	34·2	22·5	38·2	25·8	3·7% (3·5 to 3·9)	3·5% (3·2 to 3·7)
**Brazil**							
Communicable or nutritional§	17 160	3·7	3·2	5·2	3·5	1·1% (−0·1 to 2·3)	4·0% (2·7 to 5·3)
Non-communicable	46 880	9·1	10·3	12·7	11·7	−1·3% (−1·8 to −0·7)	1·0% (0·6 to 1·4)
Injuries	45 401	7·3	5·8	19·3	12·1	1·6% (1·1 to 2·1)	3·9% (3·6 to 4·2)
Ill defined	6489	1·7	1·1	2·7	1·4	2·8% (2·0 to 3·7)	5·2% (4·6 to 5·8)
All causes	115 930	21·7	20·3	39·8	28·7	0·3% (−0·3 to 0·9)	3·0% (2·7 to 3·3)
**Mexico**							
Communicable or nutritional§	9877	3·6	2·6	4·6	3·6	2·1% (1·3 to 3·0)	1·8% (1·3 to 2·2)
Non-communicable	38 483	11·8	11·2	16·5	15·8	0·7% (0·3 to 1·0)	0·5% (0·3 to 0·7)
Injuries	26 806	6·3	5·1	15·7	11·8	2·4% (1·7 to 3·1)	2·6% (2·1 to 3·0)
Ill defined	627	0·2	0·1	0·4	0·2	2·1% (0·0 to 4·2)	6·5% (5·1 to 7·8)
All causes	75 793	21·9	19·1	37·2	31·4	1·4% (1·0 to 1·8)	1·6% (1·5 to 1·7)

IGME=Inter-agency Group for Child Mortality Estimation.

* We calculated 3-year forward-moving averages of age-specific cause-specific death rates from country-level data. India data were only available for 2005–13. The remaining years were extrapolated forward using moving averages. UN IGME adjusted age-specific and sex-specific cause-specific death rates were then standardised to the WHO standard population.

† Data are the average annual rates of reduction. These are estimated by the log-linear relationship between mortality rates and year. Negative values for annual reductions indicate an increasing trend.

‡ Median all-cause deaths estimated from UN IGME were split for boys and girls using the sex distribution of death rates; population totals for each country were adopted from the UN World Population Prospects 2017 estimates.

§ The vast majority of communicable or nutritional deaths arose from infectious conditions, with malnutrition being an uncommon underlying cause of death.

¶ The cause distributions for all countries were adjusted to all-cause deaths from the UN IGME 2018.

|| For China, ill-defined causes were redistributed to other causes prior to 2013 but accounted for 2% or less of deaths, and annual reductions only reflect trends for 2013–16.

**Figure 1:**
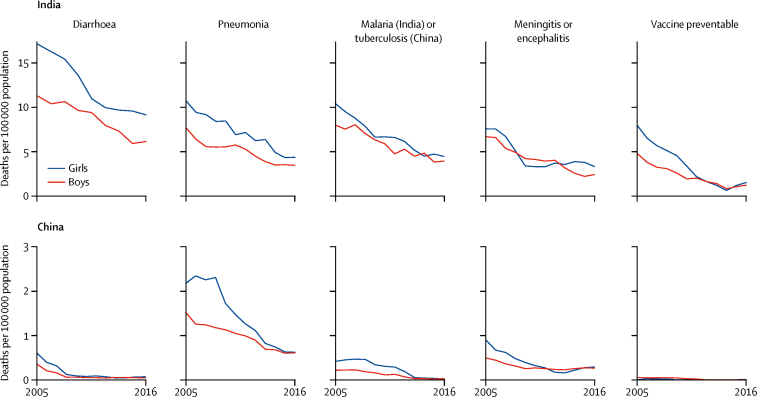
Declines in select communicable causes of death in India and China for children aged 5–14 years, 2005–16 China had substantially lower death rates from communicable causes so the *y*-axis scale is one third of the scale presented for India; in particular, death rates for malaria were very low in China so tuberculosis is shown instead. Death rates for the communicable causes considered in Brazil and Mexico were very low (appendix).

In India, the average annual rates of reduction for diarrhoea, pneumonia, malaria, meningitis or encephalitis, and vaccine-preventable conditions were similar between girls and boys (figure 1; appendix). A somewhat faster decline among girls in vaccine-preventable deaths led to similar death rates by sex by 2016 (1·5 per 100 000 girls and 1·2 per 100 000 boys). Prior to 2008, girls accounted for about three fifths of vaccine-preventable deaths but this proportion fell to two fifths by 2013. Measles deaths accounted for more than half of vaccine-preventable deaths in both sexes (data not shown). By contrast, the death rates for diarrhoea and pneumonia in 2016 were higher in girls than in boys (figure 1; table 2). In the other countries, death rates from most communicable causes were less than two deaths per 100 000 population (appendix). China showed faster declines in pneumonia deaths for girls than for boys (13·0% [11·0–15·1] for girls *vs* 8·2% [7·0–9·4] for boys; figure 1), reaching similar levels in both sexes by 2016. Pneumonia death rates in Brazil and Mexico peaked roughly in 2010 but did not differ between sexes (or between 5–9 year olds and 10–14 year olds; appendix).

**Table 2:** Major and leading causes of death for children aged 5–14 years in India, China, Brazil, and Mexico in 2016

		**Girls**		**Boys**	
		Deaths (%)	Standardised death rate per 100 000*	Deaths (%)	Standardised death rate per 100 000*
**India (population 119·7 million girls and 133·6 million boys)**					
Communicable or nutritional†		39 000 (51%)	32·6	34 920 (42%)	26·2
Non-communicable		16 100 (21%)	13·4	18 480 (22%)	13·8
Injuries		18 890 (25%)	15·8	27 770 (33%)	20·8
Leading causes					
	Cancer	2690 (4%)	2·2	3655 (4%)	2·7
	Diarrhoea	10 930 (14%)	9·1	8170 (10%)	6·1
	Drowning	4490 (6%)	3·8	8150 (10%)	6·1
	Malaria	5320 (7%)	4·5	5250 (6%)	3·9
	Meningitis or encephalitis	3970 (5%)	3·3	3210 (4%)	2·4
	Pneumonia	5210 (7%)	4·4	4600 (6%)	3·4
	Transport accidents	3180 (4%)	2·7	6460 (8%)	4·8
All causes‡		76 690 (100%)	64·1	83 640 (100%)	62·6
**China (population 75·2 million girls and 87·4 million boys)**					
Communicable or nutritional†		1280 (8%)	1·7	1310 (6%)	1·5
Non-communicable		7620 (45%)	10·2	7770 (35%)	8·9
Injuries		7800 (46%)	10·4	13 170 (59%)	15·1
Leading causes					
	Cancer	3210 (19%)	4·3	3140 (14%)	3·6
	Drowning	2600 (15%)	3·5	6130 (27%)	7·0
	Falls	640 (4%)	0·9	980 (4%)	1·1
	Neurological	1290 (8%)	1·7	1540 (7%)	1·8
	Transport accidents	2680 (16%)	3·6	3620 (16%)	4·1
All causes‡		16 930 (100%)	22·5	22 500 (100%)	25·8
**Brazil (population 15·2 million girls and 15·8 million boys)**					
Communicable or nutritional†		490 (16%)	3·2	550 (12%)	3·5
Non-communicable		1570 (51%)	10·3	1860 (41%)	11·8
Injuries		890 (28%)	5·8	1950 (43%)	12·1
Leading causes					
	Cancer	500 (16%)	3·3	650 (14%)	4·1
	Drowning	130 (4%)	0·9	370 (8%)	2·3
	Homicide	180 (6%)	1·1	510 (11%)	3·1
	Neurological	340 (11%)	2·2	410 (9%)	2·6
	Pneumonia	170 (6%)	1·1	200 (4%)	1·2
	Transport accidents	340 (11%)	2·2	600 (13%)	3·8
All causes‡		3120 (100%)	20·3	4570 (100%)	28·7
**Mexico (population 11·2 million girls and 11·7 million boys)**					
Communicable or nutritional†		300 (14%)	2·6	420 (11%)	3·6
Non-communicable		1250 (59%)	11·2	1860 (50%)	15·8
Injuries		580 (27%)	5·1	1390 (38%)	11·8
Leading causes					
	Cancer	450 (21%)	4·0	690 (19%)	5·9
	Cardiovascular	130 (6%)	1·1	180 (5%)	1·6
	Drowning	50 (2%)	0·5	190 (5%)	1·6
	Neurological	200 (9%)	1·8	340 (9%)	2·9
	Suicide	90 (4%)	0·8	150 (4%)	1·2
	Transport accidents	200 (9%)	1·7	460 (13%)	3·9
All causes‡		2140 (100%)	19·1	3700 (100%)	31·4

IGME=Inter-agency Group for Child Mortality Estimation. WPP=World Population Prospects.

* IGME adjusted age-specific and cause-specific death rates per 100 000 population were calculated using the reported population in the UN WPP 2017 and age-standardised to the WHO standard population.

† The vast majority of communicable or nutritional deaths arose from infectious conditions, with malnutrition being an uncommon underlying cause of death.

‡ Median all-cause deaths from UN IGME were split for boys and girls using the sex distribution of death rates and population totals for each country were adopted from the UN WPP 2017 estimates. Note ill-defined deaths are not shown but are included in the all cause totals.

**Figure 2:**
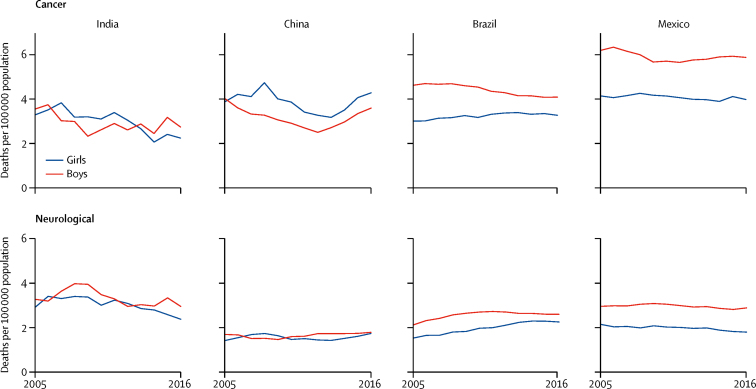
Cancer and neurological causes of death in India, China, Brazil, and Mexico for children aged 5–14 years, 2005–16

Death rates from NCDs and injuries in India were generally higher for children aged 5–14 years than in the other countries (table 1; appendix). Boys in Mexico had the highest death rate from NCDs (table 1), of which cancer accounted for more than a third (figure 2). Cancer death rates fell modestly (<2%) or remained stable across countries, except among girls in India (decline of 4·4%, 95% CI 2·5–6·3; figure 2). The proportion of cancer deaths due to leukaemia, lymphoma, and other blood cancers (ICD-10 codes C81–96) was about 30% in India, 45% in China, 43% in Brazil, and 56% in Mexico. Death rates from neurological causes in Mexico declined, but by 2016 boys had about 60% higher death rates than girls (2·9 deaths *vs* 1·8 deaths per 100 000 population; figure 2). Death rates from neurological causes in Brazil were also higher for boys than for girls, and rates for both sexes rose during the study period. China had stable and the lowest death rates from neurological causes, whereas India began with the highest death rates but they declined during the study period. Epilepsy-related deaths (G40–41) accounted for 55% of the neurological deaths in India, but only 14% of those in Brazil and 24% of those in Mexico. Cerebral palsy (G80), hydrocephalus (G91), and other brain disorders (G93) accounted for 67% of the neurological deaths in Brazil and 61% of those in Mexico.

**Figure 3:**
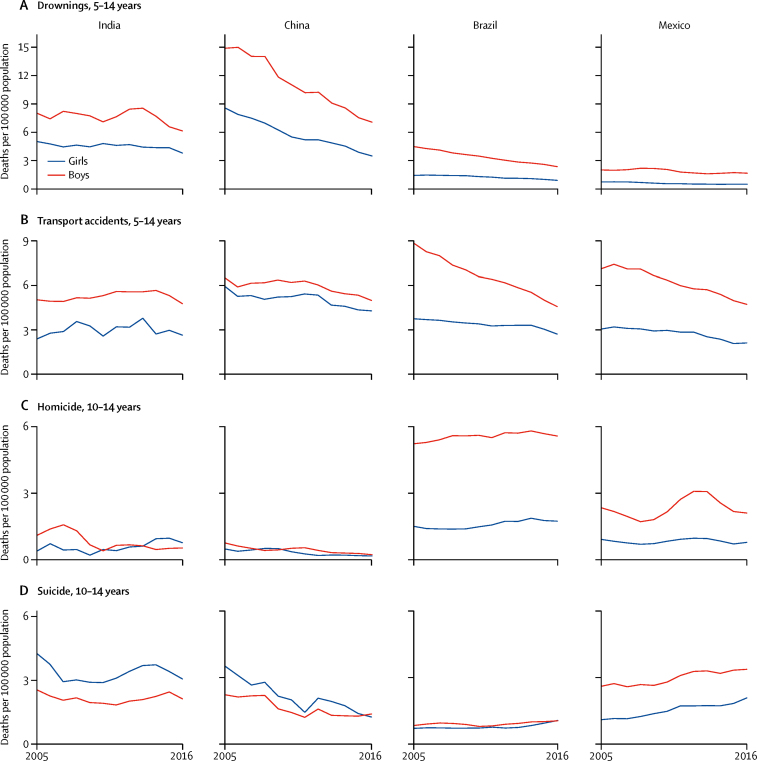
Select injury causes of death in India, China, Brazil, and Mexico for children aged 5–14 years or 10–14 years, 2005–16

Boys aged 5–14 years had higher death rates than girls for all injuries, except for suicide in both India and China (figure 3). In China, injuries accounted for the greatest proportion of deaths (table 1). Drowning death rates in China declined relatively quickly for both boys and girls compared with the other countries, but death rates remained twice as high for boys as for girls throughout the study period (figure 3). Drowning death rates in India remained unchanged, with higher rates in boys than in girls, whereas the death rates fell notably in Brazil and somewhat in Mexico. Death rates from accidents declined in China in both sexes and more rapidly for boys than for girls in Brazil and Mexico (figure 3). Where the mode of accident was specified, about 90% of the road traffic accident deaths (V01–89) in India throughout the study period occurred among vulnerable road users—defined as pedestrians, pedal cyclists, or occupants of two-wheeled or three-wheeled vehicles. This proportion of vulnerable road users in Brazil and Mexico was just below half of road traffic accidents over the same period. In China, about 57% of road traffic accident deaths occurred among pedestrians.

Nearly all deaths from homicide and suicide were among children aged 10–14 years. Death rates from homicide in Brazil and Mexico were 2–3 times higher in boys than in girls (figure 3C), with peak rates in Mexico in 2012, mostly because of firearm-related homicide (appendix). Firearm-related mortality accounted for 75% of cumulative homicide deaths for boys and 56% for girls in Brazil, with death rates for boys increasing by an average of 0·7% (95% CI 0·3–1·1) per year. In Mexico, firearm-related mortality accounted for 57% of cumulative homicide deaths for boys and 34% for girls (appendix). In India and China, the suicide death rates were higher for girls than for boys at ages 10–14 years. By contrast, in Mexico it was substantially higher for boys than for girls, increasing annually by an average of 2·8% (2·0–3·6). Suicide rates rose annually for both sexes in Brazil and Mexico but very few of these deaths were firearm related (figure 3; appendix). Hanging was the main mode of suicide in Mexico (94% of boys and 84% of girls) and Brazil (80% of boys and 67% of girls). In India, the largest mechanisms of suicide were hanging (53% of boys and 45% of girls), poisoning (11% of boys and 40% of girls), and burning (10% of boys and 11% of girls). Suicide rates did not change in India but declined rapidly in both sexes in China (figure 3).

In 2016, the five leading causes of death accounted for between half and two thirds of all deaths among boys and girls aged 5–14 years in China, Brazil, and Mexico (table 2). By contrast, India's five leading causes accounted for only about 40% of all deaths. Transport accidents are in the top three causes for both sexes in all countries, except for Indian girls. Cancer is in the top three causes for both sexes in Mexico, Brazil, and China, but was the ninth and seventh leading cause of death for boys and girls, respectively, in India (appendix).

## Discussion

We document the trends in causes of mortality in four countries that are home to about two fifths of the global population of children aged 5–14 years. By contrast with Brazil, China, and Mexico, communicable diseases still account for nearly half of deaths in India in this age group. Fast declines among girls in communicable disease mortality have narrowed the gap with boys in India and China. In Brazil and Mexico, we found no large differences at the beginning or end of the study period in death rates between boys and girls except for higher injury death rates in boys. In 2016, India had the highest death rates in every broad category except for NCDs in boys (which were highest in Mexico). In 2016, India had about 74 000 preventable or treatable deaths from communicable diseases, representing nearly half of all the annual Indian deaths and over a third of all deaths in the four countries at these ages. In China, injuries accounted for the greatest proportions of deaths, particularly from drowning. The death rates from homicide for boys aged 10–14 years in Brazil and Mexico were mostly firearm related. Deaths from transport injuries, drowning, and cancer are among the leading causes of death in all four countries.

Despite the variability in causes of death in children aged 5–14 years across these four countries, nearly all of the 244 401 study deaths arose from preventable or treatable conditions. The Global Strategy for Women's, Children's and Adolescents' Health set indicators for children younger than 5 years and those aged 10–14 years and 10–19 years.^16^ Although a full review of all relevant interventions is beyond the scope of this paper, the Disease Control Priorities project has identified cost-effective interventions against nearly all of the conditions that kill children at ages 5–14 years (appendix).^17^ For some conditions, most notably specific communicable diseases, strategies for children under 5 years could be extended to children aged 5–14 years. For example, the Indian Government has expanded its reproductive, maternal, newborn, and child health programme to include adolescents, including establishment of adolescent-friendly clinics.^18^ Delivery platforms for children, for example for pneumonia and diarrhoea, would include the use of school-based vaccination with new antigens plus use of primary care for treatment.^19^ Moreover, standards for improving the quality of care for children aged 0–15 years have been recently updated by WHO to recognise the gap in addressing the needs of children older than 5 years and adolescents.^20^

Reducing mortality among children aged 5–14 years will require a broader set of interventions than those from the maternal and child health programmes targeting children younger than 5 years, including potentially curative treatment of several specific childhood cancers (most of which cannot be prevented, based on current knowledge).^21^ The trends in homicide among boys aged 10–14 years in Brazil and Mexico are largely due to guns, and show a similar temporal pattern to young adults.^22^ Action to reduce firearm-related deaths in children in Brazil and Mexico is also possible.^17^ Prevention of drowning is also feasible.^23^ Delivery of interventions for these causes would probably be different for primary level school children than for adolescents.^19^ Indeed, causes of death differ between children aged 5–9 years and aged 10–14 years, particularly from communicable causes and some injuries (appendix), but also between children younger than 15 years and adolescents and young adults aged 15 to 29 years (in whom road traffic injuries, suicide, and cancers feature more prominently).^24^, ^25^

Our analyses of the data sources reveal the large gaps in nationwide mortality data for low-income countries. In particular, if data were available, we would expect distinct mortality patterns in sub-Saharan Africa, where more than half of the estimated global deaths of children aged 5–14 years occur.^2^, ^26^ The low death rates at ages 5–14 years relative to the rest of the lifespan demand large, nationally representative data to document changes in mortality. Modelled estimates are necessary when medically certified causes of death are not routinely collected and can be helpful to examine global trends.^27^ Modelled estimates for overall death rates at age 10–14 years diverge sharply from recorded vital rates in Europe, which has good primary data.^28^ Investments in modelled data should not come at the expense of investment in mortality surveillance systems and the statistical capacity to use country data.^27^

Deaths in children aged 5–14 years represent another important measurement challenge. For existing systems, expansion of the size of sample registration systems and improvement of quality of civil registration and medical certification systems, paired with improved hospital-based mortality statistics, are particularly relevant to children aged 5–14 years.^8^, ^29^ The sample size of the Indian SRS, covering less than 1% of deaths, results in sparse subnational data for children in this age group. Cause of death data for China did not become representative at the provincial level until 2013.^9^ Subnational studies are needed to further understand determinants for the changing patterns of drowning and road traffic injuries.^30^, ^31^ Better specification of the mode of transport, including more standard definitions of vulnerable road users, is needed. The interventions needed to reduce homicide and suicide death rates among children in Brazil and Mexico might help to avoid premature mortality from the same causes in adolescents.^22^, ^32^

We did not have access to individual level data from China, and therefore the cause classification differed slightly from the other countries. There is uncertainty in the cause of death classification across all countries, which mostly arises from different coding practices and methodological approaches. By contrast, the uncertainty from random sampling is much smaller because Brazil and Mexico have near universal coverage and the Chinese and Indian studies are reasonably large.^1^, ^2^ Nevertheless, a strength of this study is the use of nationally representative surveys with high-quality cause specification from different regions that show consistency in the leading causes of death, most of which are preventable or treatable.

In conclusion, death rates among older children are low compared with those younger than 5 years of age, but these rates can be even lower. More generally, avoidable mortality among children aged 5–14 years offers a politically visible group in which to monitor reductions in deaths from expanded universal health coverage.^16^, ^19^, ^24^ Substantial declines in deaths in this age group are possible in many countries with cost-effective, affordable, and feasible interventions.
