# Supplementary material for: Trends in cause-specific mortality among children aged 5–14 years from 2005 to 2016 in India, China, Brazil, and Mexico: an analysis of nationally representative mortality studies
Source: Lancet. 2019 Mar 16;393(10176):1119–27. doi: 10.1016/S0140-6736(19)30220-X (PMC6418656; doi:10.1016/S0140-6736(19)30220-X)
Supplement: Supplementary appendix [file mmc1.pdf]

THE LANCET

## Supplementary appendix

This appendix formed part of the original submission and has been peer reviewed. We post it as supplied by the authors.

Supplement to: Fadel SA, Boschi-Pinto C, Yu S, et al. Trends in cause-specific mortality among children aged 5–14 years from 2005 to 2016 in India, China, Brazil, and Mexico: an analysis of nationally representative mortality studies. *Lancet* 2019; published online March 12. http://dx.doi.org/10.1016/S0140-6736(19)30220-X.

**Supplementary appendix: Trends in cause-specific mortality among children aged 5-14 years from 2005 to 2016 in India, China, Brazil, and Mexico: nationally-representative mortality studies**


| Appendix contents | Page |
|---|---|
| Table 1. UN-IGME estimates for countries with highest number of deaths for children aged 5-14 years in 2017, data availability and quality of nationally-representative cause of death data | 2 |
| Table 2. ICD-10 Classification of deaths at ages 5-14 for India, Brazil, and Mexico. All causes were used in the analyses. Trends of numbered causes are presented in the manuscript. | 3 |
| Table 3. ICD-10 Classification of deaths at ages 5-14 for aggregated data from China. Ill-defined causes were reported distinctly from 2013-2016. All causes were used in the analyses. Trends of numbered causes are presented in the manuscript. | 4 |
| Table 4. ICD-10 Sub-category classification of deaths at ages 5-14 for Road traffic injuries and firearm-related injuries for India, Brazil, and Mexico | 5 |
| Figure 1. All-cause mortality envelopes used for study countries | 6 |
| Figure 2. Declines in major COD groups for children 5-14, 2005-16 | 7 |
| Table 5. Average annual rates of reduction and 95% confidence intervals for cause-specific mortality trends from 2005-2016 for children aged 5-14 in India, China, Brazil, and Mexico | 8 |
| Figure 3. Changes in rates for diarrhoea and pneumonia by age group and sex, 2005-2016 | 9 |
| Figure 4. Changes in rates for malaria and vaccine preventable diseases by age group and sex, 2005-2016 | 10 |
| Figure 5. Changes in rates for cancer and neurological diseases by age group and sex, 2005-2016 | 11 |
| Figure 6. Changes in rates for drowning and transport injuries by age group and sex, 2005-2016 | 12 |
| Figure 7. Mode of transport among road injury deaths (V01-V89) in the last three years of the study period | 13 |
| Figure 8. Crude age-specific death rates per 100 000 population for firearm-related vs. non-firearm-related homicide and suicide deaths | 14 |
| Table 6. Changes in rank of COD for children 5-14 in India, 2005 to 2016 | 15 |
| Table 7. Changes in rank of COD for children 5-14 in China, 2005 to 2016 | 16 |
| Table 8. Changes in rank of COD for children 5-14 in Brazil, 2005 to 2016 | 17 |
| Table 9. Changes in rank of COD for children 5-14 in Mexico, 2005 to 2016 | 18 |
| Table 10. Effective interventions for common avoidable causes of death of children aged 5-14 years in India, China, Brazil, and Mexico | 19 |

| Rank | Income Category | Country | Deaths in 2017 | Nationally-representative mortality system with causes (ICD-10) |
|---|---|---|---|---|
| 1 | Middle | India | 151 657 | Sample Registration System/Million Death Study, 2001-2013, good quality |
| 2 | Middle | Nigeria | 105 285 | None |
| 3 | Low | Democratic Republic of the Congo | 63 343 | None |
| 4 | Middle | Pakistan | 49 188 | None |
| 5 | Middle | China | 38 596 | Integrated disease surveillance points and vital registration, 2004-2016, good quality |
| 6 | Low | Ethiopia | 37 749 | None |
| 7 | Low | Niger | 25 854 | None |
| 8 | Middle | Indonesia | 23 974 | Sample registration system, 2014-2016, low quality |
| 9 | Low | United Republic of Tanzania | 19 137 | None |
| 10 | Middle | Cameroon | 18 911 | None |
| 11 | Low | Uganda | 17 698 | None |
| 12 | Middle | Cote d'Ivoire | 17 571 | None |
| 13 | Middle | Angola | 14 092 | None |
| 14 | Middle | Bangladesh | 14 087 | Sample registration system, |
| 15 | Low | Burkina Faso | 13 741 | None |
| 16 | Middle | Kenya | 13 473 | None |
| 17 | Middle | Philippines | 13 389 | Vital registration, 2006-2011 available in WHO mortality database |
| 18 | Low | Mali | 12 825 | None |
| 19 | Low | Mozambique | 12 502 | None |
| 20 | Low | Chad | 11 658 | None |
| 21 | Low | Somalia | 10 330 | None |
| 22 | Low | Afghanistan | 10 072 | None |
| 23 | Middle | Sudan | 9438 | None |
| 24 | Low | Madagascar | 8859 | None |
| 25 | Middle | Egypt | 8798 | Vital registration, 2000-2015, low quality |
| 26 | Middle | Ghana | 8385 | None |
| 27 | Middle | South Africa | 8192 | Vital registration, low quality, high misclassification for injuries and HIV |
| 28 | Low | Guinea | 7844 | None |
| 29 | Middle | Myanmar | 7754 | None |
| 30 | Middle | Brazil | 7308 | Vital registration, good quality |
| 31 | Low | Malawi | 6967 | None |
| 32 | Low | Benin | 6854 | None |
| 33 | Middle | Zambia | 6656 | None |
| 34 | Low | Burundi | 6499 | None |
| 35 | Low | South Sudan | 6265 | None |
| 36 | Low | Senegal | 6197 | None |
| 37 | Low | Zimbabwe | 6151 | None |
| 38 | Middle | Mexico | 5711 | Vital registration, good quality |
| 39 | Low | Yemen | 5635 | None |
| **SUBTOTAL of above countries/Global total** | | | **818 645 / 928 587 (88%)** | |
| **SUBTOTAL of four countries included/ LMIC countries with accessible nationally-representative cause of death data (%)** | | | **203 272 / 233 651 (87%)** | |

**Appendix Table 1. UN-IGME estimates for countries with highest number of deaths for children aged 5-14 years in 2017, data availability and quality of nationally-representative cause of death data.** Income categories are based on the World Bank.[1] Completeness of coverage for death registration and causes of death for countries were reported by the World Health Organization and the United Nations Statistics Division.[2,3] We considered countries with vital registration completeness or without a nationally-representative sample registration system as not having a nationally-representative mortality system. Sample registration data from Indonesia and Bangladesh were not accessible; however quality and completeness of cause of death data are low for both systems.[4,5]

| Cause of death | ICD-10 range |
|---|---|
| **I. Communicable and nutritional conditions** | |
| 1. Pneumonia | H65-H68, H70-H71, J00-J22, J32, J36, J85-J86, P23, U04 |
| 2. Tuberculosis | A15-19, J65, B90 |
| 3. Diarrhoea | A00-A09 |
| 4. Vaccine Preventable Diseases | |
| Measles | B01, B05 |
| Other selected vaccine preventable diseases | A33, A35,A36-A37, A80, B03, B06,B26, B91 |
| 5. Meningitis/encephalitis | A39, A81, A83-A89, G00-G09 |
| 6. Malaria | B50-B54 |
| Other causes | |
| Nutritional deficiencies | D50-D53, E00-E02, E40-E46, E50-E64, X53-X54 |
| HIV | B20-B24, C46, D84, R75 |
| Acute bacterial sepsis and severe infections | A20-A28, A32, A38, A40-A49, A68, A70, A74-A79, B95-B96, H10, H60, I30, I32-I33, I39-I41, K02, K04-K05, K61, K65, K67, K81, L00-L04, L08, M00-M01, M60, M86, N10, N30, N34, N41, N49, N61, P36-P39, U80-U89 |
| Other infectious and parasitic diseases | A30, A31, A50, A51-A53, A54-A64, A65-A67, A69, A71, A82, A90-A99, B00 B02 B04 B07-B09 B15-B17 B19 B25 B27-B49 B55-B58 B59, B60/B64 B65-B83, B85-B89, B92, B94, B97-B99, N70-N74, P35, Y95 |
| Fever of unknown origin | R50 |
| Maternal and Perinatal conditions | O00-O99, A34, F53; P00-P22, P24-P29, P50-P96 |
| **II. Non-communicable diseases** | |
| 7. Cancer | |
| Leukemia, lymphoma, blood cancers | C81-C96, D45-D47 |
| Brain and CNS cancers | C69-C72, D31-D33, D42-D43 |
| Other Cancers | C00-C45, C47-C68, C73-C80, C97, D00-D30, D34-D41, D44, D48, N60, N62-N64, N87, R59 |
| 8. Neurological | G40-G41, R56, F00-F09, F11-F39, F40-F52, F54-F99, G10-G37, G43-G44, G47-G73, G80, G90-G99, R26-R29, R40-R49, R90 |
| Other Non-communicable diseases | |
| Cardiovascular | G45-G46, G81-G83, I00-I28, I31, I34-I38, I42-I84, I86-I99, R00-R01, R03, R55 |
| Diabetes, Endocrine and immune disorders | E10-E14, D55-D63, D64, D65-D83, D86-D89, E03-E07, E15-E16, E20-E35, E65-E90, R70-R74, R76, R77, R81 |
| Congenital | Q00-Q99 |
| Chronic Respiratory diseases | J30-J31, J33-J35, J37-J64, J66-J84, J90-J99, R04-R06, R84, R91 |
| Other NCDs | B18, F10, H00-H06, H11-H59, H61-H62, H69, H72-H95, I85, K00-K01, K03, K06-K14, L05, L10-L99, M02-M54, M61-M85, M87-M99, R20-R23, I85, K20-K60, K62-K63, K66, K70-K80, K82-K93, R10-R19, R63, R85, X45, Y15, Y90, Y91, N00-N08, N11-N29, N31-N33, N35-N40, N42-N48, N50-N51, N75-N86, N88-N99, R30-R39, R80, R82, R86, R87 |
| **III. Injuries** | |
| 9. Transport accidents | V01-V89, V90-V99, Y85, |
| 10. Drowning | W65-W74 |
| 11. Suicide | X60-X84 |
| 12. Homicide | X85-Y09 , Y35-Y36, Y87 |
| Other injuries | |
| Falls | W00-W19 |
| Poisonings, fires, and venomous | X40-X44, X46-X49, X00-X09, X20-X29, W57 |
| Other unintentional | W20-W56, W58-W59, W64, W75-W99, X10-X19, X30-X39, X50-X52, X57-X59, Y40-Y84, Y86, Y88-Y89 |
| All other injuries | Y10-Y14, Y16-Y34, Y96-Y98 |
| **IV. Ill-defined or cause unknown** | R02, R07-R09, R25, R51-R54, R57-R58, R60-R62, R64-R69, R78-R79, R83, R89, R92-R94, R96, R98-R99 |

**Appendix Table 2. ICD-10 Classification of deaths at ages 5-14 for India, Brazil, and Mexico.** All causes were used in the analyses. Trends of numbered causes are presented in the manuscript.

| Cause of death | China DSP ICD-10 range |
|---|---|
| **I. Communicable and nutritional conditions** | |
| 1. Diarrhoea | A00, A01, A03, A04, A06-A09 |
| 2. Pneumonia | J00-J06, J10-J18, J20-J22, H65-H66 |
| 3. Tuberculosis | A15-A19, B90 |
| 4. Vaccine Preventable Diseases | |
| Measles | B05 |
| Other selected vaccine preventable diseases | A33-A37, A80, B91 |
| 5. Meningitis/encephalitis | A39, A83.0, G00, G03 |
| 6. Malaria | B50-B54 |
| Other causes | |
| Nutritional deficiencies | E00-E02, E40-E46, E50, D50-D53, D64.9, E51-E64 |
| HIV | B20-B24 |
| Other infectious and parasitic diseases | A02, A05, A20-A28, A30-A32, A38, A40-A79, A81, A82, A83.1-A83.9, A84-A99, B00-B04, B06-B19, B25-B49, B55-B60, B64-B89, B92-B99, G04, I30-I33, I38, I40, I42, N70-N73 |
| Maternal and Perinatal conditions | O00-O99, P00-P96 |
| **II. Non-communicable diseases** | |
| 7. Cancer | |
| Leukemia, lymphoma, blood cancers | C81-C96 |
| Other Cancers | C00-C16, C18-C22, C25, C33-C34, C43-C44, C50, C53-C56, C61, C67, D00-D48 |
| 8. Neurological | F01-F99, G06-G98 |
| Other Non-communicable diseases | |
| Cardiovascular | I00-I13, I20-I28, I34-I37, I44-I51, I60-I99 |
| Diabetes, Endocrine and immune disorders | D55-D64 (minus D64.9), D65-D89, E03-E07, E10-E14, E15-E16, E20-E34, E65-E88 |
| Chronic Respiratory diseases | J30-J98 |
| Congenital | Q00-Q99 |
| Other NCDs | H00-H61, H68-H93, K00-K01, K03-K14, K20-K92, L00-L98, M00-M99, N00-N64, N75-N98 |
| **III. Injuries** | |
| 9. Transport accidents | V01-V04, V06, V09-V80, V87, V89, V99 |
| 10. Drowning | W65-W74 |
| 11. Suicide | X60-X84, Y87.0 |
| 12. Homicide | X85-Y09 , Y35-Y36, Y87.1 |
| Other injuries | |
| Falls | W00-W19 |
| Poisonings and fires | X40-X49, X00-X09 |
| Other unintentional | V05, V81-V86, V88, V90-V98, W20-W64, W75-W99, X10-X39, X50-X59, Y40-Y86, Y88, Y89 |
| **IV. Ill-defined** | R0-R99 |

**Appendix Table 3. ICD-10 Classification of deaths at ages 5-14 for aggregated data from China.** Ill-defined causes were reported distinctly from 2013-2016. All causes were used in the analyses. Trends of numbered causes are presented in the manuscript.

| Cause of death | ICD-10 |
|---|---|
| **Mode of transport for road traffic injuries** | |
| Road traffic accidents | V01-V06, V09-V80, V87, V89 |
| Pedestrian | V01-V06, V09 |
| Cyclist | V10-V19 |
| Two and three-wheeled vehicles | V20-V39 |
| Cars, vans | V40-V59 |
| Large motor vehicles | V60-V79 |
| Other or unknown mode of transport | V80-V89 |
| **Firearm-related injuries** | |
| Suicide | X60-X84 |
| Firearm-related suicide | X72-X74 |
| Homicide | X85-Y09 , Y35-Y36, Y87.1 |
| Firearm-related homicide | X93-X95 |
| Unintentional firearm discharge | W32-W34 |

**Appendix Table 4. ICD-10 Sub-category classification of deaths at ages 5-14 for Road traffic injuries and firearm-related injuries for India, Brazil, and Mexico.**

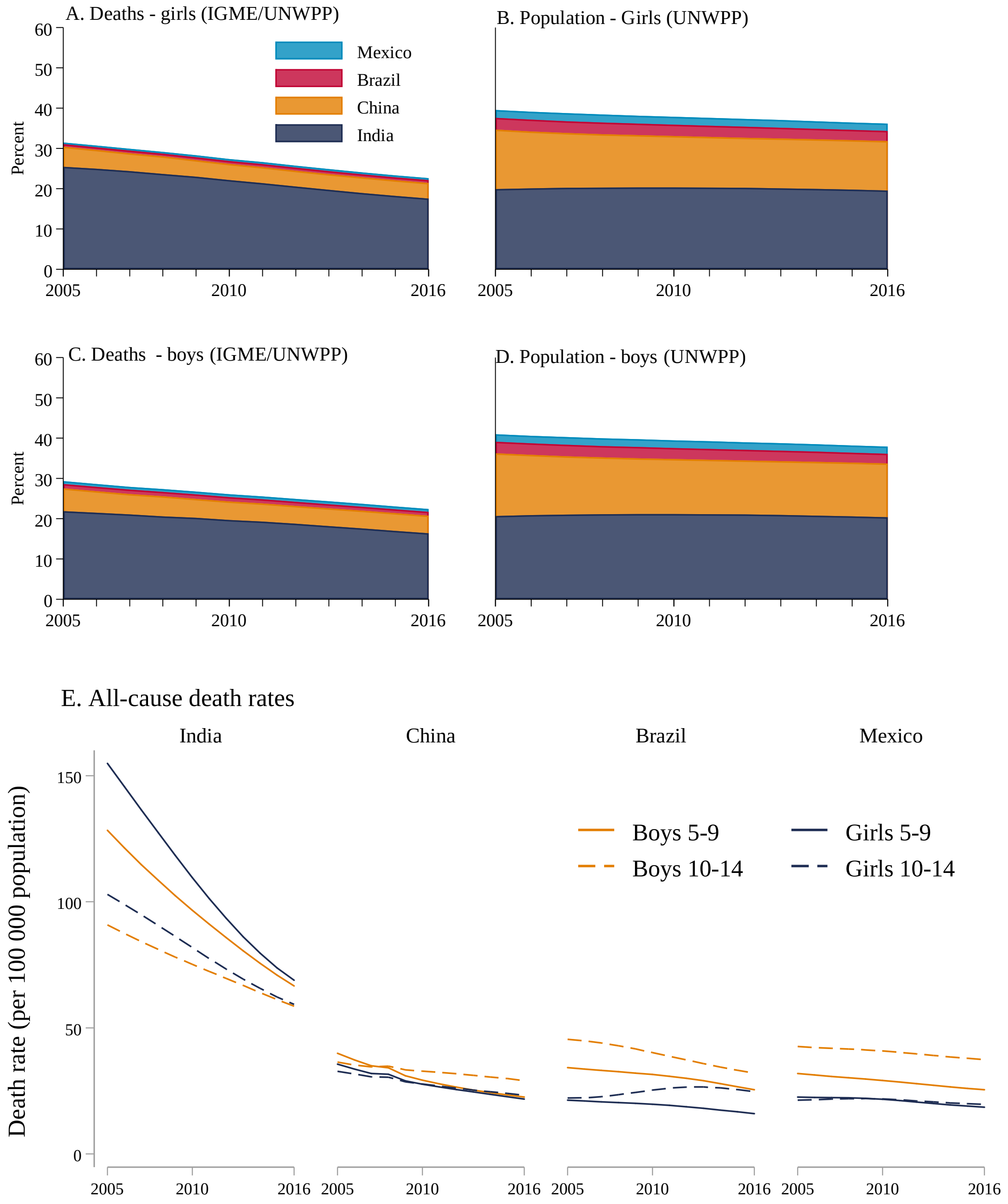


**Appendix Figure 1**. **All-cause mortality envelopes used for study countries.** A, C. Percent of global deaths and B, D. percent of global populations for girls (A-B) and boys (C-D) represented by the study countries' deaths and populations, respectively. E. The contribution of total deaths in all four countries to global deaths declined from 30% in 2005 to 23% in 2016. All cause death rates for children 5-9 and 10-14 by sex. UN-IGME estimated deaths were used to calculate death rates for 5-9 and 10-14 year olds. Sex ratios for each 5-year age group were calculated by dividing the sex-specific central mean death rates by those for both sexes retrieved directly from UNWPP. The sex ratios were applied to IGME age-specific rates to calculate sex and age-specific rates and counts. UNWPP mortality rates for girls 10-14 years in Brazil were lower for 2005-2010 than for 2010-2015 due to an increase in probability of dying reported by IBGE in their life tables for 2011, but do not reflect a significant increase in mortality trend across all the years.

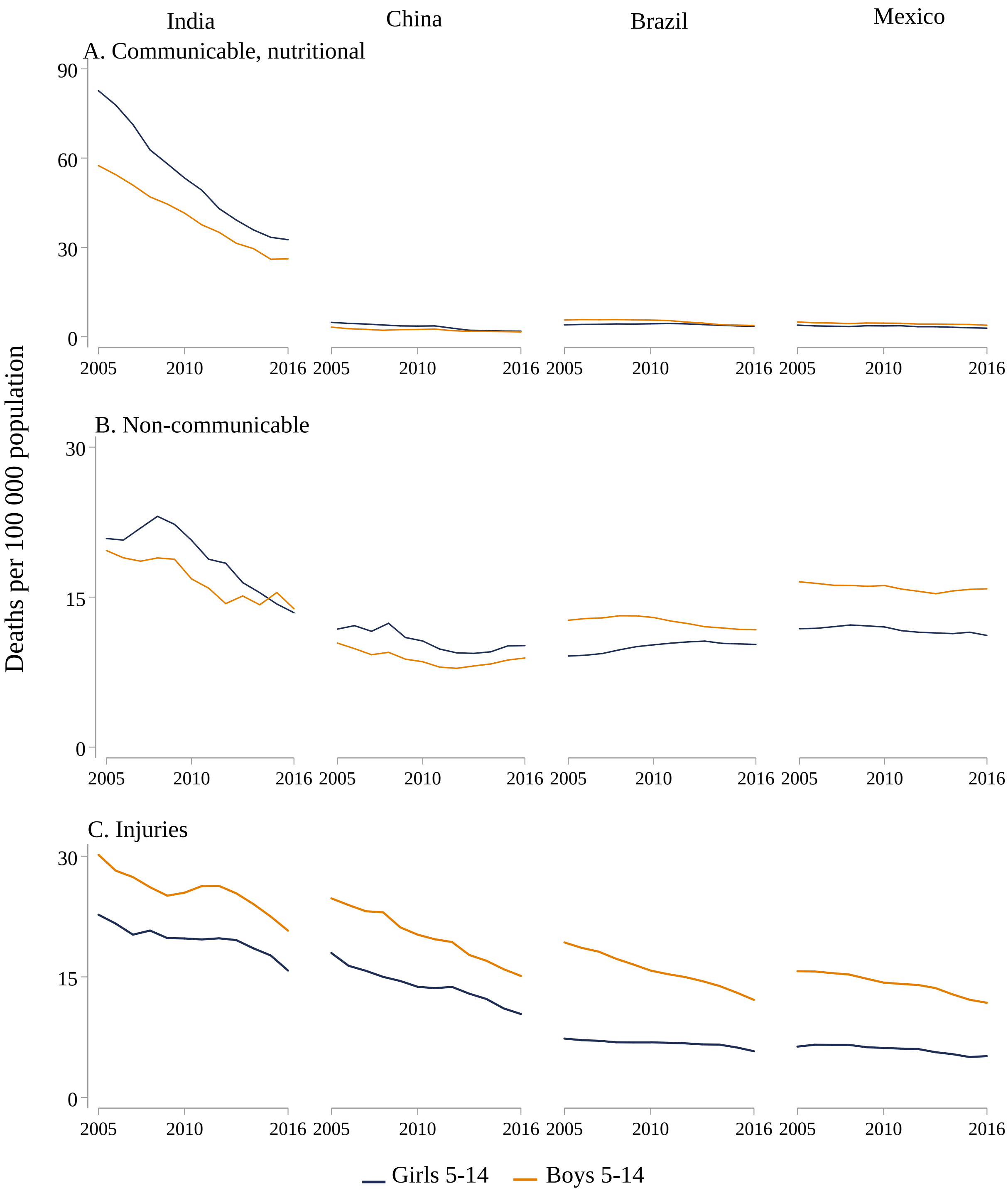


**Appendix Figure 2. Declines in major COD groups for children 5-14, 2005-16**

| Cause of death | India | | China | | Brazil | | Mexico | |
|---|---|---|---|---|---|---|---|---|
| | Girls | Boys | Girls | Boys | Girls | Boys | Girls | Boys |
| Communicable, nutritional | 8·7 (8·2, 9·1) | 7·4 (6·9, 7·8) | 8·9 (7·3, 10·6) | 5·2 (3·7, 6·7) | 1·1 (-0·1, 2·3) | 4·0 (2·7, 5·3) | 2·1 (1·3, 3·0) | 1·8 (1·3, 2·2) |
| Diarrhoea | 8·6 (7·5, 9·6) | 7·5 (6·5, 8·6) | 19·0 (11·7, 25·7) | 14·8 (7·4, 21·6) | 3·5 (0·4, 6·5) | 2·7 (0·1, 5·1) | 5·5 (3·7, 7·3) | 1·9 (0·6, 3·3) |
| Pneumonia | 7·9 (6·7, 9·0) | 6·7 (5·3, 8·1) | 13·0 (11·0, 15·1) | 8·2 (7·0, 9·4) | -1·1 (-2·5, 0·3) | 2·2 (1·0, 3·4) | 1·0 (-1·9, 3·9) | 2·0 (0·1, 3·8) |
| Tuberculosis | 15·2 (12·4, 17·9) | 1·7 (-1·3, 4·6) | 26·7 (19·5, 33·2) | 22·5 (17·6, 27·2) | 3·6 (1·8, 5·4) | 5·9 (4·0, 7·8) | 0·9 (-2·3, 4·1) | 4·0 (1·0, 6·9) |
| Meningitis/encephalitis | 7·0 (3·2, 10·6) | 9·5 (8·1, 11·0) | 11·9 (6·4, 17·0) | 5·4 (2·4, 8·2) | 3·6 (2·1, 5·1) | 5·1 (2·6, 7·5) | 3·1 (1·2, 5·0) | 3·2 (2·1, 4·3) |
| Malaria | 7·6 (6·6, 8·6) | 6·9 (5·6, 8·2) | | | 11·6 (4·2, 18·5) | 12·5 (7·2, 17·5) | | |
| Vaccine-preventable | 18·8 (13·7, 23·6) | 13·5 (10·8, 16·1) | 15·7 (4·5, 25·6) | 13·4 (3·5, 22·3) | -2·0 (-4·6, 0·5) | 1·4 (-2·0, 4·7) | 12·9 (9·7, 16·0) | 9·8 (7·4, 12·1) |
| Other communicable, nutritional | 7·0 (5·6, 8·3) | 6·9 (5·7, 8·1) | -12·4 (-23·3, -2·4) | -8·9 (-16·2, -2·1) | 1·5 (0·3, 2·6) | 5·2 (4·0, 6·3) | 0·6 (-0·2, 1·3) | 0·6 (-0·1, 1·4) |
| Non-communicable | 4·4 (2·9, 5·9) | 3·3 (2·4, 4·2) | 2·3 (1·1, 3·4) | 1·6 (0·4, 2·8) | -1·3 (-1·8, -0·7) | 1·0 (0·6, 1·4) | 0·7 (0·3, 1·0) | 0·5 (0·3, 0·7) |
| Cancer | 4·4 (2·5, 6·2) | 1·9 (-0·4, 4·2) | 1·1 (-1·2, 3·2) | 1·5 (-1·0, 4·0) | -1·0 (-1·4, -0·5) | 1·5 (1·1, 1·8) | 0·4 (0·1, 0·8) | 0·6 (0·0, 1·2) |
| Neurological | 2·4 (1·0, 3·9) | 1·6 (-0·2, 3·3) | -0·2 (-1·7, 1·3) | -1·1 (-2·2, 0·0) | -4·0 (-4·6, -3·4) | -1·5 (-2·6, -0·4) | 1·3 (0·8, 1·8) | 0·5 (0·1, 0·9) |
| Cardiovascular | 6·4 (3·3, 9·4) | 3·7 (-1·6, 8·8) | -5·8 (-9·0, -2·8) | -3·5 (-4·9, -2·0) | 0·9 (0·2, 1·6) | 2·8 (1·8, 3·7) | -3·7 (-4·8, -2·5) | -1·8 (-2·6, -1·0) |
| Other non-communicable | 4·7 (2·4, 6·9) | 4·2 (3·0, 5·4) | 5·4 (4·8, 6·1) | 4·3 (3·4, 5·2) | -0·9 (-1·8, 0·0) | 1·3 (0·9, 1·6) | 1·5 (1·1, 1·9) | 1·0 (0·4, 1·6) |
| Injuries | 2·3 (1·6, 3·1) | 2·4 (1·6, 3·3) | 4·2 (3·6, 4·8) | 4·4 (4·0, 4·8) | 1·6 (1·1, 2·1) | 3·9 (3·6, 4·2) | 2·4 (1·7, 3·1) | 2·6 (2·1, 3·0) |
| Transport accidents | -0·6 (-3·3, 2·0) | -0·6 (-1·7, 0·4) | 2·3 (1·3, 3·3) | 1·9 (1·0, 2·8) | 2·3 (1·6, 3·0) | 5·4 (5·0, 5·9) | 3·8 (2·6, 4·9) | 4·0 (3·4, 4·6) |
| Drowning | 1·4 (0·5, 2·4) | 1·3 (-0·4, 3·0) | 7·5 (6·8, 8·2) | 7·0 (6·2, 7·7) | 4·3 (3·4, 5·1) | 5·7 (5·4, 5·9) | 4·7 (3·7, 5·8) | 2·5 (1·2, 3·9) |
| Falls | 1·3 (-1·5, 4·1) | 2·2 (-1·6, 5·8) | 4·3 (1·9, 6·7) | 3·9 (1·9, 5·8) | 6·3 (4·6, 7·9) | 6·9 (6·1, 7·7) | 3·3 (1·1, 5·4) | 2·5 (0·0, 4·9) |
| Suicide | -0·5 (-3·5, 2·4) | 0·0 (-1·6, 1·5) | 7·8 (5·8, 9·8) | 5·8 (3·9, 7·7) | -2·8 (-4·3, -1·3) | -1·3 (-2·4, -0·1) | -5·9 (-7·0, -4·9) | -2·8 (-3·6, -2·0) |
| Homicide | -1·0 (-4·2, 2·0) | 10·7 (6·7, 14·6) | 9·1 (7·4, 10·7) | 11·5 (9·2, 13·6) | -2·5 (-3·4, -1·5) | -0·4 (-0·9, 0·1) | -0·5 (-2·3, 1·2) | -1·6 (-4·9, 1·6) |
| Other injuries | 4·9 (4·3, 5·5) | 5·1 (4·2, 6·0) | -5·8 (-9·7, -2·0) | -8·7 (-12·9, -4·8) | 1·4 (-0·2, 2·9) | 3·6 (2·9, 4·3) | 3·5 (1·9, 5·0) | 3·8 (3·3, 4·3) |

**Appendix Table 5. Average annual rates of reduction and 95% confidence intervals for cause-specific mortality trends from 2005-2016 for children aged 5-14 years in India, China, Brazil, and Mexico.**

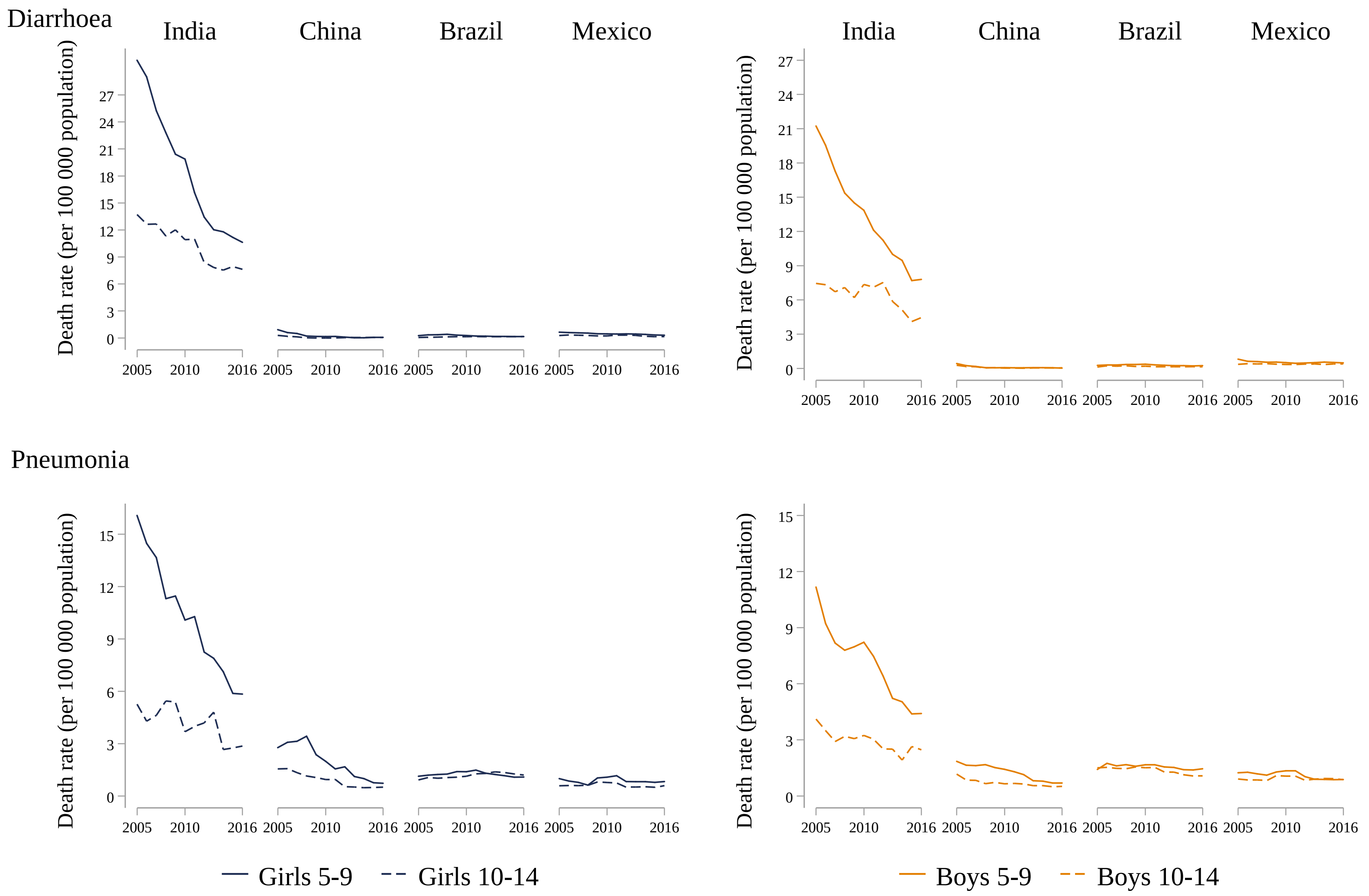


**Appendix Figure 3. Changes in rates for diarrhoea and pneumonia by age group and sex, 2005-2016**

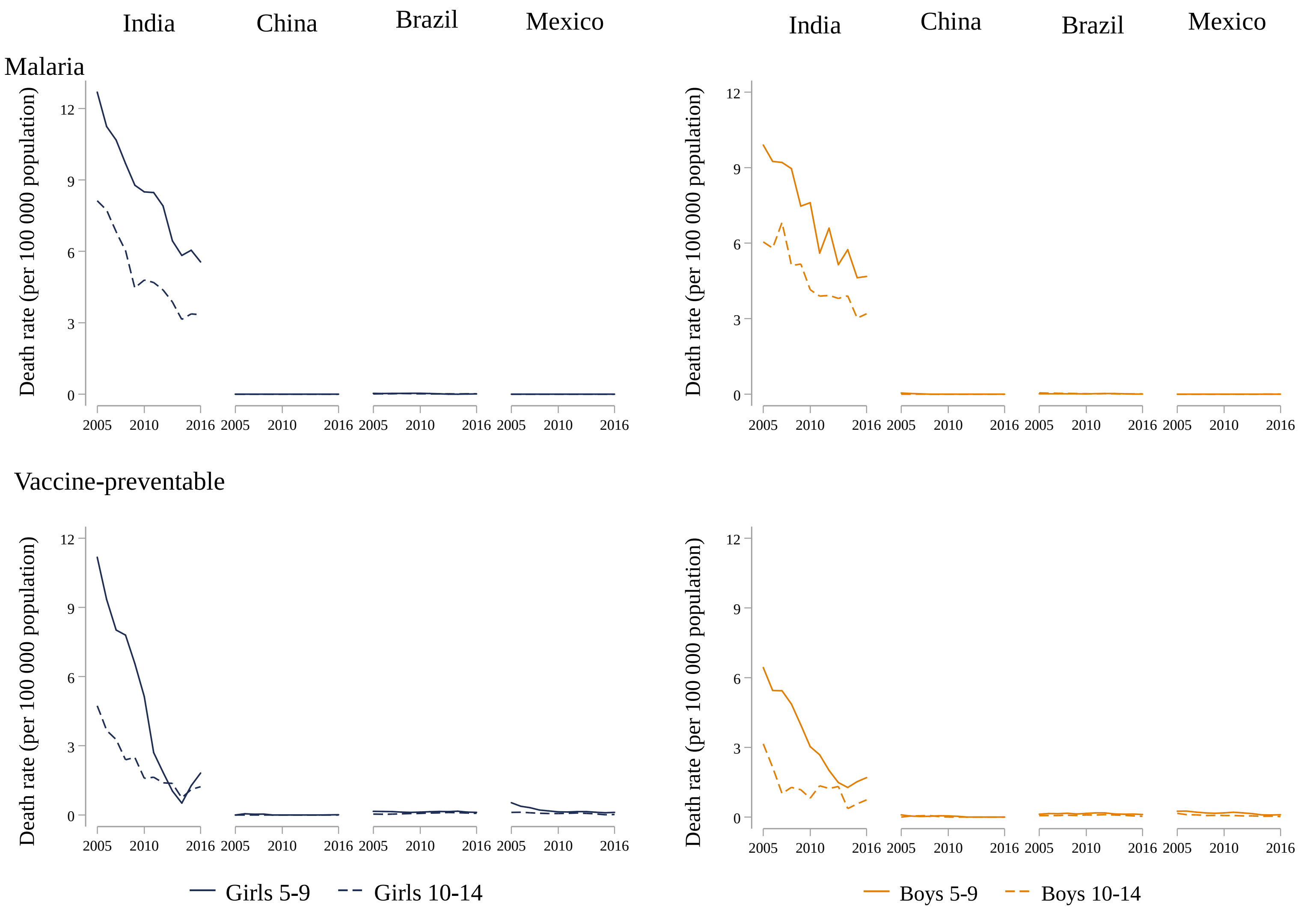


**Appendix Figure 4. Changes in rates for malaria and vaccine preventable diseases by age group and sex, 2005-2016**

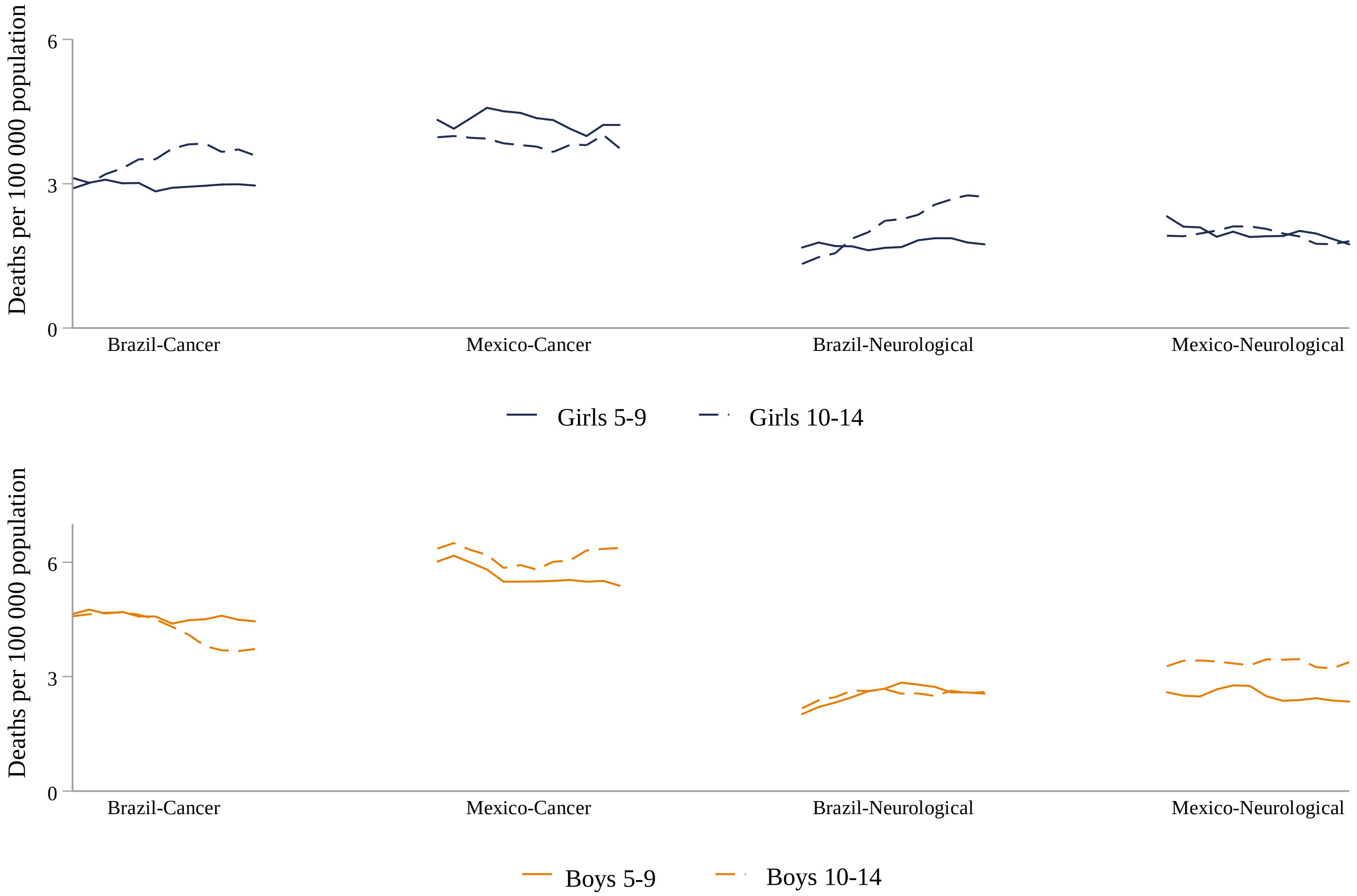


**Appendix Figure 5. Changes in rates for cancer and neurological diseases by age group and sex, 2005-2016**

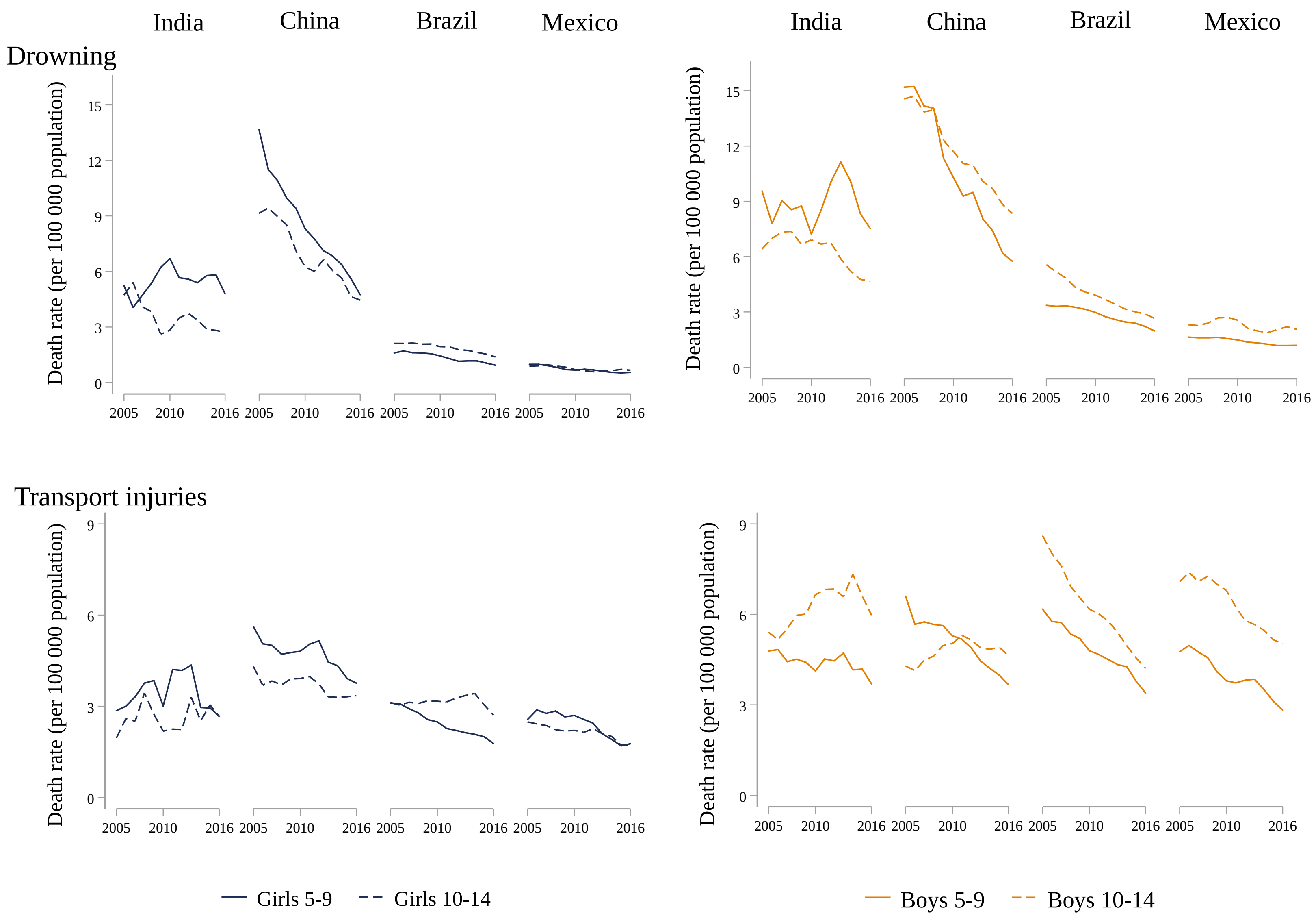


**Appendix Figure 6. Changes in rates for drowning and transport accidents by age group and sex, 2005-2016**

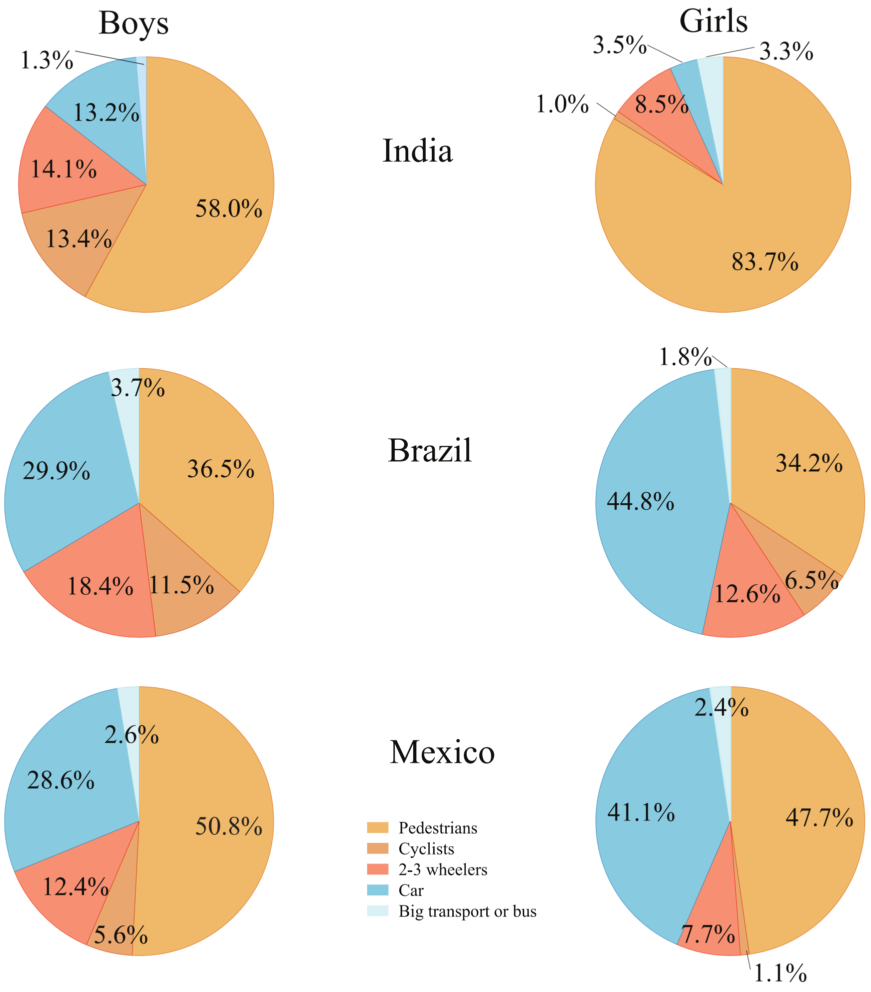


**Appendix Figure 7. Mode of transport among road injury deaths (V01-V89) in the last three years of the study period.** Vulnerable road users are pedestrians, cyclists, and occupants of two-three wheeled motor vehicles. Unspecified mode of transport were excluded and assumed to not differ from the distribution when mode of transport was specified. These were much higher in Brazil (boys 23.2%, girls 18.7%) and Mexico (boys 40.4%, girls 43.9%), than in India (boys 12.8%, girls 1.7%). For China, only pedestrian and car related accidents were reported among road traffic accidents from 2013-2016. Pedestrians accounted for 57% of boys and 59% of girls among these deaths. Cars accounted for 11% of road traffic injury deaths for boy and girls in China.

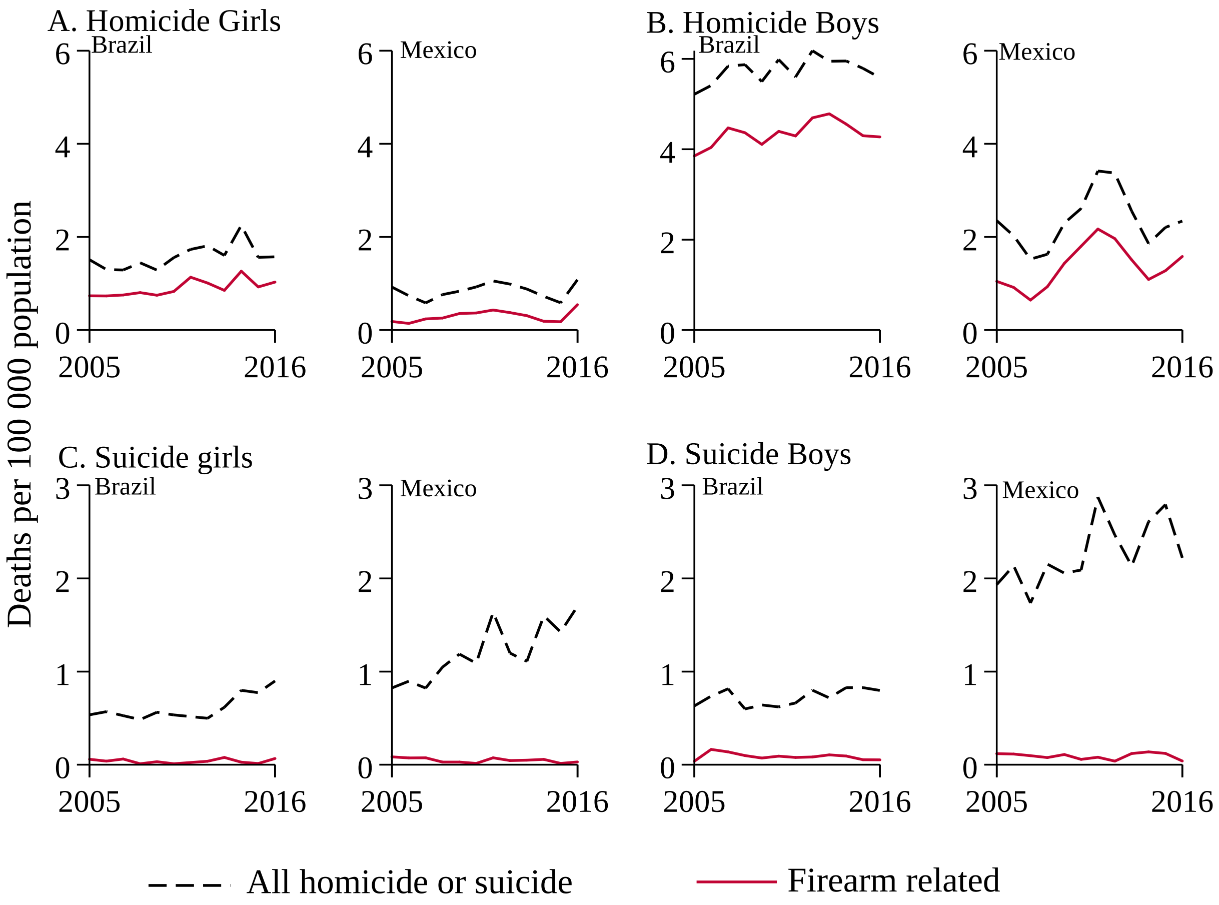


**Appendix Figure 8. Crude age-specific death rates per 100 000 population for firearm-related homicide (A-B) and suicide (C-D) deaths for girls (A, C) and boys (B, D) 10-14 aged years in Brazil and Mexico, 2005-16.** Homicide rates increased annually an average of 0.7% (0.3%-1.1%) for boys in Brazil. In Mexico, homicide death rates peaked in 2012, but then decreased to rates 2.5 times lower than for Brazil. Suicide death rates were also increasing twice as fast annually in Mexico (girls 5.9%, 7.0%-4.9%; boys 2.8%, 3.6%-2.0%) than in Brazil (girls 2.8%, 1.2%- 4.4%; boys 1.4%, 0.03%-2.73%). Suicide rates were two times higher for boys than for girls in Mexico. The mode of suicide was mostly hanging for both countries. Deaths due to unintentional firearm discharge accounted for 3% and 12% of firearm deaths in Brazil and Mexico, respectively.

| Sex, Cause and Death rate per 100 000 population (2005) | | % of all cause deaths |
|---|---|---|
| **Girls** | | |
| 1. Diarrhoea | 22·3 | 17·3 |
| 2. Pneumonia | 10·7 | 8·3 |
| 3. Malaria | 10·4 | 8·1 |
| 4. Vaccine-preventable* | 8·0 | 6·2 |
| 5. Meningitis or encephalitis | 7·6 | 5·9 |
| All other defined causes | | 52·0 |
| Ill-defined | 2·8 | 2·2 |
| **All causes** | 129·0 | |
| | | |
| **Boys** | | |
| 1. Diarrhoea | 14·4 | 13·2 |
| 2. Drowning | 8·0 | 7·3 |
| 3. Malaria | 8·0 | 7·3 |
| 4. Pneumonia | 7·7 | 7·0 |
| 5. Meningitis or encephalitis† | 6·7 | 6·1 |
| All other defined causes | | 57·0 |
| Ill-defined | 2·3 | 2·1 |
| **All causes** | 109·7 | |

| Sex, Cause and Death rate per 100 000 population (2016) | | % of all cause deaths |
|---|---|---|
| **Girls** | | |
| 1. Diarrhoea | 9·1 | 14·3 |
| 2. Malaria | 4·5 | 6·9 |
| 3. Pneumonia | 4·4 | 6·8 |
| 4. Drowning* | 3·8 | 5·9 |
| 5. Meningitis or encephalitis | 3·3 | 5·2 |
| All other defined causes | | 57·5 |
| Ill-defined | 2·3 | 3·5 |
| **All causes** | 64·1 | |
| | | |
| **Boys** | | |
| 1. Diarrhoea | 6·1 | 9·8 |
| 2. Drowning | 6·1 | 9·7 |
| 3. Transport accidents† | 4·8 | 7·7 |
| 4. Malaria | 3·9 | 6·3 |
| 5. Pneumonia | 3·4 | 5·5 |
| All other defined causes | | 58·0 |
| Ill-defined | 1·9 | 3·0 |
| **All causes** | 62·6 | |

**Appendix Table 6. Changes in rank of COD for children 5-14 in India, 2005 to 2016**

*For girls aged 5-14 years in India, vaccine-preventable deaths were the fourth leading cause of death in 2005 but changed to tenth leading cause in 2016. Drowning changed from the seventh leading cause in 2005 to fourth leading cause in 2016.

† For boys aged 5-14 years in India, transport accidents changed from sixth leading cause of death in 2005 to the third leading cause of death in 2016. Meningitis or encephalitis declined from fifth leading cause of death in 2005 to eighth leading cause of death in 2016

| Sex, Cause and Death rate per 100 000 population (2005) | | % of all cause deaths |
|---|---|---|
| **Girls** | | |
| 1. Drowning | 8·6 | 24·5 |
| 2. Transport accidents | 5·0 | 14·4 |
| 3. Cancer | 3·9 | 11·6 |
| 4. Pneumonia* | 2·2 | 6·1 |
| 5. Suicide* | 1·3 | 4·3 |
| All other defined causes | | 39·1 |
| Ill-defined | | |
| **All causes** | 34·2 | |
| | | |
| **Boys** | | |
| 1. Drowning | 14·9 | 39·1 |
| 2. Transport accidents | 5·4 | 14·0 |
| 3. Cancer | 4·0 | 10·6 |
| 4. Falls | 1·7 | 4·5 |
| 5. Neurological | 1·7 | 4·3 |
| All other defined causes | | 27·5 |
| Ill-defined | | |
| **All causes** | 38·2 | |

| Sex, Cause and Death rate per 100 000 population (2016) | | % of all cause deaths |
|---|---|---|
| **Girls** | | |
| 1. Cancer | 4·3 | 19·0 |
| 2. Transport accidents | 3·6 | 15·8 |
| 3. Drowning | 3·5 | 15·3 |
| 4. Neurological* | 1·7 | 7·6 |
| 5. Falls | 0·9 | 3·8 |
| All other defined causes | | 37·1 |
| Ill-defined | 0·3 | 1·4 |
| **All causes** | 22·5 | |
| | | |
| **Boys** | | |
| 1. Drowning | 7·0 | 27·2 |
| 2. Transport accidents | 4·1 | 16·1 |
| 3. Cancer | 3·6 | 14·0 |
| 4. Neurological | 1·8 | 6·8 |
| 5. Falls | 1·1 | 4·3 |
| All other defined causes | | 30·4 |
| Ill-defined | 0·3 | 1·1 |
| **All causes** | 25·8 | |

**Appendix Table 7. Changes in rank of COD for children 5-14 in China, 2005 to 2016.**

*For girls aged 5-14 years in China, pneumonia was the fourth leading cause of death in 2005 but changed to seventh leading cause in 2016. Suicide changed from the fifth leading cause in 2005 to eighth leading cause in 2016. Neurological causes increased from sixth leading cause in 2005 to fourth leading cause in 2016

| **Sex, Cause and Death rate per 100 000 population (2005)** | | **% of all cause deaths** |
|---|---|---|
| **Girls** | | |
| 1. Transport accidents | 3·1 | 14·3 |
| 2. Cancer | 3·0 | 13·8 |
| 3. Neurological | 1·5 | 6·9 |
| 4. Drowning* | 1·4 | 6·4 |
| 5. Cardiovascular* | 1·2 | 5·3 |
| All other defined causes | | 45·5 |
| Ill-defined | 1·7 | 7·6 |
| **All causes** | 21·7 | |
| | | |
| **Boys** | | |
| 1. Transport accidents | 7·4 | 18·6 |
| 2. Cancer | 4·6 | 11·6 |
| 3. Drowning | 4·5 | 11·2 |
| 4. Homicide | 3·0 | 7·5 |
| 5. Neurological | 2·1 | 5·3 |
| All other defined causes | | 39·2 |
| Ill-defined | 2·7 | 6·7 |
| **All causes** | 39·8 | |

| **Sex, Cause and Death rate per 100 000 population (2016)** | | **% of all cause deaths** |
|---|---|---|
| **Girls** | | |
| 1. Cancer | 3·3 | 16·0 |
| 2. Transport accidents | 2·2 | 11·0 |
| 3. Neurological | 2·2 | 11·0 |
| 4. Homicide* | 1·1 | 5·7 |
| 5. Pneumonia | 1·1 | 5·6 |
| All other defined causes | | 45·4 |
| Ill-defined | 1·1 | 5·3 |
| **All causes** | 20·3 | |
| | | |
| **Boys** | | |
| 1. Cancer | 4·1 | 14·1 |
| 2. Transport accidents | 3·8 | 13·2 |
| 3. Homicide | 3·1 | 11·2 |
| 4. Neurological | 2·6 | 8·9 |
| 5. Drowning | 2·3 | 8·1 |
| All other defined causes | | 39·7 |
| Ill-defined | 1·4 | 4·7 |
| **All causes** | 28·7 | |

**Appendix Table 8. Changes in rank of COD for children 5-14 in Brazil, 2005 to 2016**

*For girls aged 5-14 years in Brazil, drowning was the fourth leading cause of death in 2005 but changed to seventh leading cause in 2016. Cardiovascular diseases changed from the fifth leading cause in 2005 to sixth leading cause in 2016. Homicide was seventh leading cause in 2005 and fourth leading cause in 2016.

| **Sex, Cause and Death rate per 100 000 population (2005)** | | **% of all cause deaths** |
|---|---|---|
| **Girls** | | |
| 1. Cancer | 4·1 | 18·9 |
| 2. Transport accidents | 2·5 | 11·5 |
| 3. Neurological | 2·1 | 9·7 |
| 4. Pneumonia* | 0·8 | 3·6 |
| 5. Cardiovascular | 0·8 | 3·5 |
| All other defined causes | | 52·1 |
| Ill-defined | 0·2 | 0·7 |
| **All causes** | 21·9 | |
| | | |
| **Boys** | | |
| 1. Cancer | 6·2 | 16·6 |
| 2. Transport accidents | 5·9 | 15·9 |
| 3. Neurological | 2·9 | 7·9 |
| 4. Drowning | 2·0 | 5·3 |
| 5. Homicide† | 1·5 | 4·1 |
| All other defined causes | | 49·1 |
| Ill-defined | 0·4 | 1·1 |
| **All causes** | 37·2 | |

| **Sex, Cause and Death rate per 100 000 population (2016)** | | **% of all cause deaths** |
|---|---|---|
| **Girls** | | |
| 1. Cancer | 4·0 | 20·8 |
| 2. Neurological | 1·8 | 9·3 |
| 3. Transport accidents | 1·7 | 9·1 |
| 4. Cardiovascular | 1·1 | 5·9 |
| 5. Suicide | 0·8 | 4·1 |
| All other defined causes | | 50·0 |
| Ill-defined | 0·1 | 0·7 |
| **All causes** | 19·1 | |
| | | |
| **Boys** | | |
| 1. Cancer | 5·9 | 18·7 |
| 2. Transport accidents | 3·9 | 12·5 |
| 3. Neurological | 2·9 | 9·1 |
| 4. Drowning | 1·6 | 5·2 |
| 5. Cardiovascular† | 1·6 | 5·0 |
| All other defined causes | | 48·8 |
| Ill-defined | 0·2 | 0·7 |
| **All causes** | 31·4 | |

**Appendix Table 9. Changes in rank of COD for children 5-14 in Mexico, 2005 to 2016**

*For girls aged 5-14 years in Mexico, pneumonia was the fourth leading cause of death in 2005 but changed to sixth leading cause in 2016.

† For boys aged 5-14 years in Mexico, Cardiovascular diseases replaced homicide deaths as the fifth leading cause of death in 2016. Homicide was the sixth leading cause of death for boys in 2016.

| Cause of death | Estimated deaths in four study countries, 2016 | SDG Ref. No. | Disease Control Priorities Interventions |
|---|---|---|---|
| Drowning | 22 110 | | Parental or other adult supervision; Swimming lessons |
| Diarrhoea | 19 320 | 3.3, 3.9 | Rotavirus vaccination; Early use of oral rehydration solutions<br>Appropriate use of antibiotics for bloody diarrhoea and dysentery<br>Nutritional interventions for persistent diarrhoea; Rapid restoration of nutritional status |
| Road traffic injuries | 17 550 | 3.6 | Separating different types of road users (e.g. motor vehicles from pedestrians)<br>Increasing visibility of pedestrians and cyclists; Reducing average speeds through traffic calming measures; Setting and enforcing blood alcohol concentration limits<br>Setting and enforcing use of seat belts for all motor vehicle occupants |
| Cancer | 14 980 | 3.4 | Twinning programs between hospitals in LMIC and established cancer treatment centers<br>Treatment of early-stage childhood cancers (e.g. Burkitt and Hodgkin lymphoma, acute lymphoblastic leukemia) with curative intent in pediatric cancer units or hospitals |
| Pneumonia | 11 360 | 3.3 | Community-level pneumococcal vaccination; Pneumococcal vaccine every five years for those with underlying lung disease; Community-administered oral antibiotics for treatment |
| Suicide | 4780 | 3.4 | Restricting availability of toxic pesticides and other commonly used methods; organizing community-based safe storage activities for pesticides, other poisons, and medications<br>Reducing the availability and excessive use of alcohol and illicit drugs; Decriminalization of suicide to de-stigmatize reporting; Working with national and local media organizations to limit inappropriate reporting of suicides; Reducing the stigma associated with suicide and mental disorders to encourage help-seeking behavior; Brief interventions for people who have attempted suicide; Improving medical management of poisoning associated with high case-fatality |
| Epilepsy | 4500 | 3.4 | Managing epilepsy patients, including acute stabilization and long-term management with generic anti-epileptics |
| Tuberculosis (TB) | 4100 | 3.3 | Routine contact tracing to identify individuals exposed to TB and linking them to care; Referring cases of TB treatment failure for drug susceptibility testing; enrolling those with MDR-TB for treatment per WHO guidelines; Using rapid molecular diagnostics (UltraXpert), including assessment of rifampicin resistance, to diagnose TB; Initiating first-line treatment per current WHO guidelines for drug-susceptible TB; referral for confirmation, further assessment of drug resistance, and treatment of drug-resistant TB<br>Screening for latent TB infection following a new diagnosis of HIV, followed by yearly screening among PLHIV at high risk of TB exposure; initiation of isoniazid preventive therapy among all individuals who screen positive but do not have evidence of active TB<br>Systematic identification of individuals with TB symptoms among high-risk groups and linkage to care ("active case finding")<br>Specialized TB services, including management of MDR- and XDR-TB treatment failure and surgery for TB |
| Homicide | 2530 | 16.1 | Interventions for problem drinkers; Parent training for high-risk families, including nurse home visitation for child maltreatment; Gun control |
| All causes | 213 270 | | |

**Appendix Table 10. Effective interventions for common avoidable causes of death of children aged 5-14 years in India, China, Brazil, and Mexico.** Interventions were compiled from the Disease Control Priorities volumes.[6]

**Appendix references**


1. The World Bank. World Bank Country Classification by income. 2018. https://datahelpdesk.worldbank.org/knowledgebase/articles/906519-world-bank-country-and-lending-groups; (accessed December 27, 2018)
2. World Health Organization. SDG Target 17.19|Death registration. 2018. http://apps.who.int/gho/data/node.sdg.17-19-data?lang=en (accessed December 27, 2018)
3. United Nations Statistics Division. 2017. Coverage of Birth and Death Registration. https://unstats.un.org/unsd/demographic-social/crvs/ (accessed December 27, 2018)
4. Usman Y, Iriawan RW, Rosita T, et al. Indonesia's Sample Registtration System in 2018: A work in progress. Journal of Population and Social Studies 2019;27:39-52.
5. Managment Information System and Directorate General of Health Services. Report of the Comprehensive Assessment & Strategic Action Plan on Civil Registration & Vital Statistics System in Bangladesh. Dhaka: MOHFW; 2013.
6. Jamison DT, Nugent R, Gelband H, Horton S, Jha P, Laxminarayan R, eds. Disease control priorities, 3rd edn. Washington, DC: World Bank, 2015.
